# Discovery of Isoplumbagin as a Novel NQO1 Substrate and Anti-Cancer Quinone

**DOI:** 10.3390/ijms21124378

**Published:** 2020-06-19

**Authors:** Yen-Chi Tsao, Yu-Jung Chang, Chun-Hsien Wang, Linyi Chen

**Affiliations:** 1 Institute of Molecular Medicine, National Tsing Hua University, Hsinchu 30013, Taiwan; u91062702@gmail.com (Y.-C.T.); wang841115@gmail.com (C.-H.W.); 2 Department of Medical Science, National Tsing Hua University, Hsinchu 30013, Taiwan

**Keywords:** isoplumbagin, NQO1, quinone, cancer, metastasis

## Abstract

Isoplumbagin (5-hydroxy-3-methyl-1,4-naphthoquinone), a naturally occurring quinone from *Lawsonia inermis* and *Plumbago europaea*, has been reported to have anti-inflammatory and antimicrobial activity. Inflammation has long been implicated in cancer progression. In this study, we examined the anticancer effect of chemically synthesized isoplumbagin. Our results revealed that isoplumbagin treatment suppressed cell viability and invasion of highly invasive oral squamous cell carcinoma (OSCC) OC3-IV2 cells, glioblastoma U87 cells, non-small cell lung carcinoma H1299 cells, prostate cancer PC3 cells, and cervical cancer HeLa cells by using 3-(4,5-dimethylthiazol-2-yl)-2,5-diphenyltetrazolium bromide (MTT) and Boyden chamber assays. In vivo studies demonstrate the inhibitory effect of 2 mg/kg isoplumbagin on the growth of orthotopic xenograft tumors derived from OSCC cells. Mechanistically, isoplumbagin exerts its cytotoxic effect through acting as a substrate of reduced nicotinamide adenine dinucleotide phosphate [NAD(P)H] dehydrogenase quinone 1 (NQO1) to generate hydroquinone, which reverses mitochondrial fission phenotype, reduces mitochondrial complex IV activity, and thus compromises mitochondrial function. Collectively, this work reveals an anticancer activity of isoplumbagin mainly through modulating mitochondrial dynamics and function.

## 1. Introduction

Medicinal plants and their metabolites are great sources for pharmaceutical applications. The metabolites in plants provide a rich variety of bioactive compounds with anticancer, antioxidant, anti-inflammatory, or antimicrobial activities. Some of these drugs such as paclitaxel, docetaxel, resveratrol, vincristine, and vinblastine are approved of and used extensively in treating several types of cancer, including breast, head and neck, testicular, and bladder cancers [1,2]. Natural quinones are secondary metabolites of plant and are categorized as benzoquinone, naphthoquinone, phenanthrenequinone, and anthraquinone according to their aromatic carbon skeleton [3]. Quinones are highly electrophilic molecules that accept one- or two-electrons from flavoenzymes and iron-sulfur proteins to form semiquinone or hydroquinone. They exert cytotoxic effects through alkylating proteins or DNA and affect the redox cycle with their semiquinone radicals to generate reactive oxygen species. Their cytotoxicity promotes inflammatory reactions, oxidizes DNA, and induces cell death. Quinone-based drugs such as doxorubicin and mitomycin C have been used clinically for cancer chemotherapy, but their adverse side effects and toxicity have been an issue [4]. Consequently, there is a continued search for the development of quinone-based agents displaying antitumor activity that are less toxic and have reduced side effects.

Isoplumbagin (5-hydroxy-3-methyl-1,4-naphthoquinone) can be isolated from the bark of *Lawsonia inermis* [5] and *Plumbago europaea* [6] and has been shown to exhibit anti-inflammatory activity against Carrageenan-induced rat paw oedema [7] and antimicrobial activity against invasive vaginitis strains [6]. Chronic inflammation facilitates the initiation and progression of cancer [8]. Although isoplumbagin is known to exert pharmaceutical effects, no report thus far demonstrates its anticancer effect. Therefore, this study aims to evaluate its potential anticancer effect and the underlying mechanism.

## 2. Results and Discussion

### 2.1. Isoplumbagin Suppresses Proliferation and Invasion of Cancer Cells

**Figure 1:**
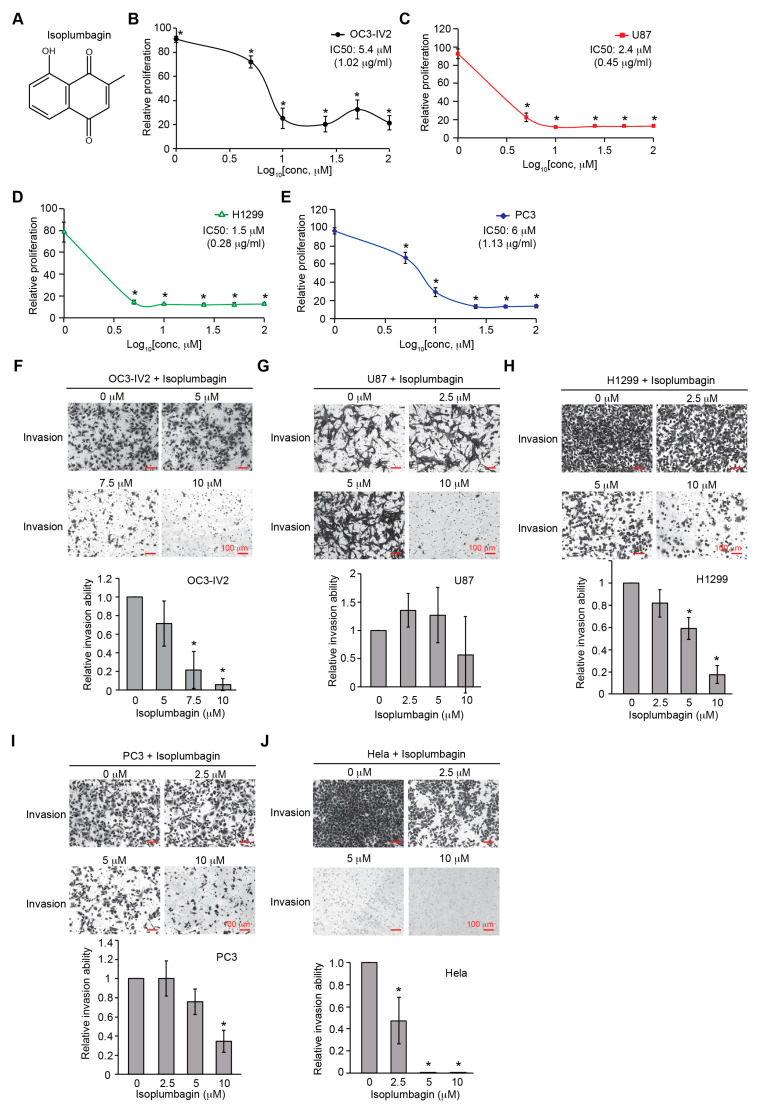
The chemical structure of isoplumbagin and its effect on survival and invasion of cancer cells: (**A**) Chemical structure of isoplumbagin. (**B**–**E**) The 3-(4,5-dimethylthiazol-2-yl)-2,5-diphenyltetrazolium bromide (MTT) assay was used to determine proliferation of OC3-IV2 (**B**), U87 (**C**), H1299 (**D**), and PC3 (**E**) cells treated with isoplumbagin for 48 h. (**F**–**J**) Invasion of OC3-IV2 (**F**), U87 (**G**), H1299 (**H**), PC3 (**I**), and HeLa (**J**) cells treated with isoplumbagin was assessed with the Boyden chamber assays. Values for invasion were normalized to those for cells treated with solvent control (dimethyl sulfoxide, DMSO). * Compared with solvent control (DMSO). Data from three independent experiments are presented as mean ± S.E.M. (* *p* < 0.05, paired Student’s *t*-test). Scale bar: 100 μm.

In this study, isoplumbagin was chemically synthesized with 95% purity from AKos GmbH Company (Figure S1). The effect of isoplumbagin (Figure 1A) on cell survival among various cancer cell lines was evaluated by 3-(4,5-dimethylthiazol-2-yl)-2,5-diphenyltetrazolium bromide (MTT) assays. OC3-IV2, in vivo-selected for highly metastasized oral squamous cell carcinoma (OSCC) cells [9]; U87 (glioblastoma); H1299 (non-small cell lung carcinoma); and PC3 (prostate cancer) cells were treated with 0, 1, 5, 10, 25, 50, and 100 μM of isoplumbagin for 48 h. The growth of OC3-IV2 and PC3 cells was inhibited below 25–30% by isoplumbagin treatment at a concentration of 10–25 μM, and the IC50 was 5.4 μM for OC3-IV2 and 6 μM for PC3 cells. In contrast, isoplumbagin treatment inhibited cell survival of U87 and H1299 cells to reach 80–85% suppression at a concentration of less than 5 μM, and the IC50s were 2.4 μM and 1.5 μM, respectively, displaying stronger antiproliferative activities of isoplumbagin against U87 and H1299 cells compared to that of OC3-IV2 and PC3 cells (Figure 1B–E). To examine whether isoplumbagin affects cancer cell invasion by Boyden chamber assays, as shown in Figure 1F–J, 5 to 10 μM of isoplumbagin was sufficient to suppress the invasion of OC3-IV2, H1299, PC3, and human cervical cancer HeLa cells but not U87 cells. These results reveal differential sensitivity of these cancer cells to isoplumbagin in terms of survival and invasion. U87 and H1299 cell survival are more sensitive to isoplumbagin, whereas invasion of OC3-IV2 and HeLa cells is largely inhibited by isoplumbagin.

### 2.2. Isoplumbagin Inhibits Tumor Growth in an OSCC Xenograft Model

To test the anticancer effect of isoplumbagin in vivo, the orthotopic xenograft model was established by injecting OC3-IV2 cells to oral buccal of CB17/lcr-Prkdc^scid^/CrlNarl mice. When the tumor volume in oral buccal reached up to 25 mm^3^, isoplumbagin was intraperitoneally injected once every three days for 2 weeks. 

**Figure 2:**
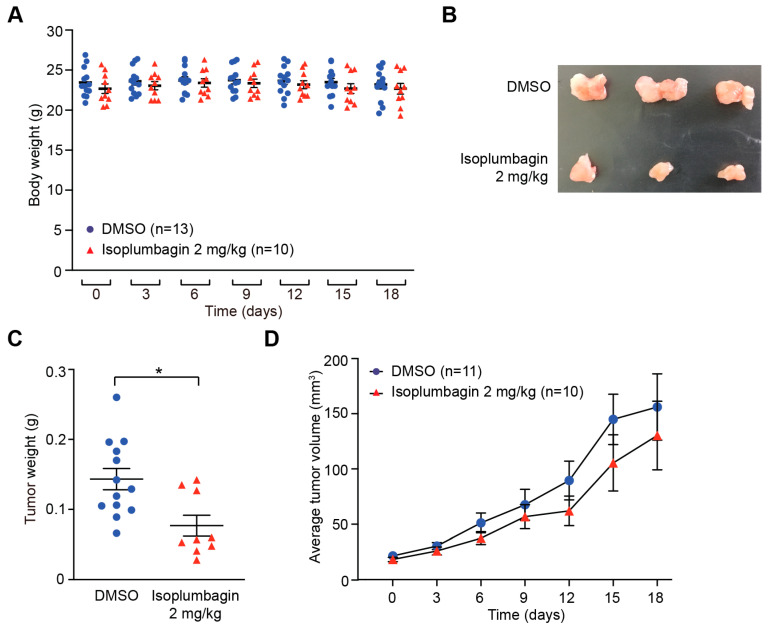
The effect of isoplumbagin in an orthotopic xenograft mice model: (**A**) OC3-IV2 cells were injected into the oral buccal mucosa of CB17/lcr-Prkdc^scid^/CrlNarl mice. After tumor volume reached approximately 25 mm^3^, mice were treated with vehicle (DMSO, *n* = 13) or isoplumbagin (2 mg/kg, *n* = 10) via intraperitoneal injections once every 3 days. Body weights of mice were measured from days 0–18 after treatment. Each value in the graph represents the mean ± S.E.M from mice. (**B**–**C**) Mice were sacrificed, and tumors were isolated from the vehicle (DMSO, *n* = 13) or isoplumbagin treatment (*n* = 9) at 18 days. Representative photograph of tumors are shown in (**B**). The tumor weight (**C**) of harvested tumor was measured from (**B**). Each value in the graph represents the mean ± S.E.M from harvested tumor (* *p* < 0.05, Student’s *t*-test). (**D**) Tumor volumes from (**A**) with vehicle- (DMSO, *n* = 11) or isoplumbagin-treated (*n* = 10) mice were measured during the course of the 18-day treatment. Statistical analysis of results in (**D**) was carried out using the one-way ANOVA. Each value in the graph represents the mean ± S.E.M.

The body weights of isoplumbagin- and vehicle-treated mice were similar (Figure 2A), indicative of no obvious systemic toxicity at this treatment regimen. On the other hand, the average tumor weights for mice treated with 2 mg/kg of isoplumbagin were reduced (Figure 2B,C) and the tumor sizes in mice treated with isoplumbagin were approximately 50% smaller compared to control mice. During the course of the 18-day treatment regimen, the average tumor volume in 10 mice treated with 2 mg/kg of the isoplumbagin groups was decreased compared to the vehicle-treated 11 mice (Figure 2D). Due to the difficulty of measuring tumor volume in the small oral cavity of mice, the variation of tumor volume is relatively large, rendering no statistical difference. Nonetheless, these in vivo data indicate that 2 mg/kg isoplumbagin inhibits the growth of OSCC-derived tumors without systemic toxicity.

### 2.3. Isoplumbagin Is a Substrate of NAD(P)H Dehydrogenase Quinone 1 (NQO1)

**Figure 3:**
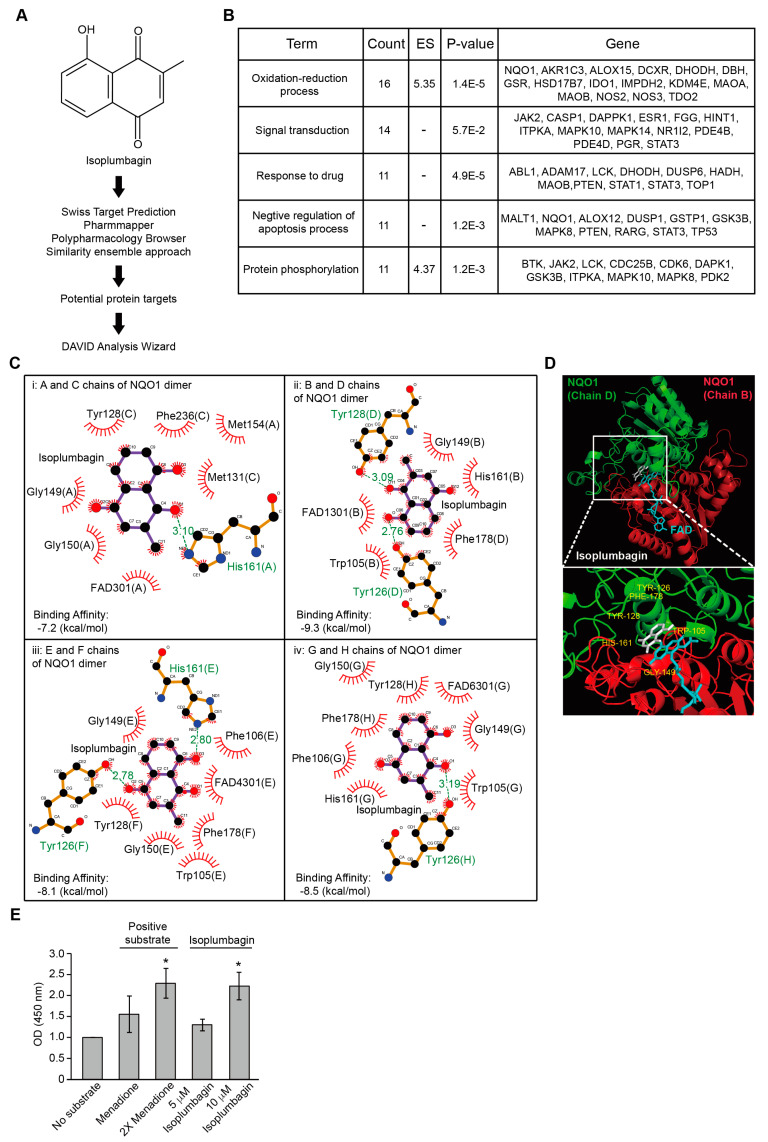
Isoplumbagin is a substrate of reduced nicotinamide adenine dinucleotide phosphate [NAD(P)H] dehydrogenase quinone 1 (NQO1). (**A**) The predicted targets of isoplumbagin. (**B**) The gene ontology term enrichment results of potential binding targets for isoplumbagin based on the Database for Annotation, Visualization, and Integrated Discovery (DAVID) analysis. (**C**) Schematic representation of isoplumbagin and NQO1 homodimer interaction: i for A and C chains, ii for B and D chains, iii for E and F chains, and iv for G and H chains of NQO1 homodimers. Flavin adenine dinucleotide (FAD) is a cofactor of NQO1. The calculated binding affinity of isoplumbagin and NQO1 homodimer is shown. Hydrogen bonds and van der Waals interactions are shown in stick representation and decorated arc, respectively. (**D**) The molecular docking between isoplumbagin and the B/D chains of NQO1: Isoplumbagin is colored in white; the NQO1 homodimer is colored in red for chain B and in green for chain D; and FAD is in blue. (**E**) The NQO1 enzyme activity was calculated in the presence of menadione or isoplumbagin. * Compared with control (no substrate). Data from three independent experiments are presented as mean ± S.E.M. (* *p* < 0.05, paired Student’s *t*-test).

To identify the candidate targets for isoplumbagin, prediction software was used by the Swiss target prediction, pharmmapper, polypharmacology browser, and similarity ensemble approach, followed by the Database for Annotation, Visualization, and Integrated Discovery (DAVID) to investigate of the interaction network (Figure 3A). The top five functions of isoplumbagin-targeted proteins were related to the oxidation–reduction process, signal transduction, response to drug, negative regulation of apoptosis process, and protein phosphorylation (Figure 3B). Based on the cluster and the highest enrichment score, the NQO1 protein was ultimately chosen for further investigation. Physically, NQO1 enzyme functions as a homodimer [10,11] that uses the reduced nicotinamide adenine dinucleotide (NADH) or reduced nicotinamide adenine dinucleotide phosphate (NADPH) as a cofactor to catalyze the reduction of quinones to hydroquinones. To understand how isoplumbagin interacts with the NQO1 homodimer, the molecular docking and ligand binding simulation approach was used to examine their probable interaction. Four combinations of NQO1 homodimer are shown based on eight chains of crystal structure of NQO1 [Protein Data Bank (PDB) code 2F1O]. Isoplumbagin bound to the enzymatic active site of NQO1 homodimers through hydrogen bonds with residues Tyr126, Tyr128, or His161 and van der Waals interactions with other residues Trp105, Phe106, Met131, Gly149, Gly150, Met154, His161, Phe178, and Phe236 of NQO1 (Figure 3C). Based on their predicted binding affinity and the distance of hydrogen bonds, we propose that the most stable complex is isoplumbagin and the B/D chains of NQO1 (Figure 3D). Compared to the previously reported interaction between NQO1 and its inhibitor dicoumarol, residues Trp105, Tyr126, Tyr128, Gly149, His161, and Phe178 of NQO1 were involved [12]. To further investigate whether isoplumbagin is a substrate or an inhibitor of NQO1 using an in vitro NQO1 activity assays, we used menadione in this assay as a substrate of NQO1, together with cofactor NADH, and reduced menadione for further reduction of water-soluble tetrazolium salt to yellow color formazan. As shown in Figure 3D, formazan was increased in the presence of menadione compared to no substrate, similar to the effect of isoplumbagin. This result suggests that isoplumbagin is an NQO1 substrate.

### 2.4. NQO1 Levels Vary in Different Types of Cancer

**Figure 4:**
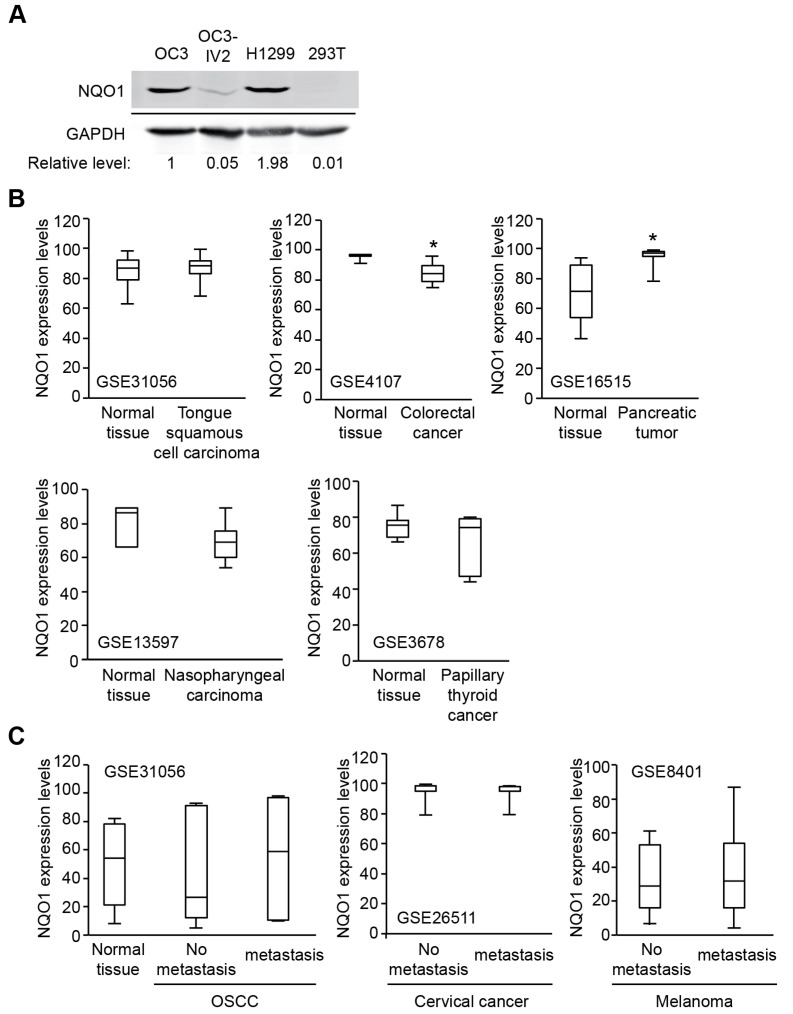
NQO1 levels in cancer cell lines and in patient tissues: (**A**) Cell lysates of OC3, OC3-IV2, H1299, and 293T cells were resolved via SDS-PAGE followed by immunoblotting with anti-NQO1 and anti-glyceraldehyde 3-phosphate dehydrogenase (GAPDH) antibodies. The relative level of NQO1 was normalized to the GAPDH level. (**B**) Gene expression data obtained from the Gene Expression Omnibus (GEO) database were used to analyze NQO1 expression in tumor and normal tissues. (**C**) The gene expression of NQO1 in tumor and metastasis from GEO database is shown. * Compared with respective normal tissues (* *p* < 0.05, Student’s *t*-test).

NQO1 facilitates detoxification of quinones to stable hydroquinones through a two-electron reduction process (Figure S2A). The two-electron reduction catalysed by NQO1 protects cells from oxidative stress, thereby avoiding the production of semiquinone radicals. In response to oxidative stress, activation of the Keap1/Nrf2/ARE pathway leads to induced NQO1 [13,14,15]. This mechanism has been implicated in multiple tumorigenesis processes, such as lung, pancreatic, and breast cancer, for survival [10,11], suggesting that NQO1 is a potential therapeutic target to treat cancer [12]. Interesting, NQO1 was found higher in OC3 cells and H1299 compared to normal 293T cells, but NQO1 expression was significantly reduced in highly invasive OC3-IV2 cells compared with its parental OC3 cells (Figure 4A). In addition, analysis of public Gene Expression Omnibus (GEO) datasets for NQO1 expression in patient samples of various cancer types showed different expression profiles in primary tumors compared with their respective normal tissues or metastatic stage. NQO1 expression was decreased in colorectal cancer and nasopharyngeal carcinoma; was increased in pancreatic tumor and metastatic OSCC; and no different in tongue squamous cell carcinoma, papillary thyroid cancer, and metastatic stage of cervical cancer and melanoma (Figure 4B,C). These results suggest that high or low NQO1 might not be a bona fide prognostic marker. Nonetheless, lower NQO1 expression correlates with advanced prostate cancer, metastatic tissues, and mesenchymal attributes [16]. Thus, targeting NQO1 should carefully consider the stage/progress of the diseases.

Dicoumarol, an available NQO1 inhibitor which competes with NAD(P)H for binding to NQO1, had no obvious effect on the suppression of OSCC growth compared to isoplumbagin (Figures S1B and S3A). This phenomenon implies that inhibition of NQO1 by dicoumarol is not a useful approach for the treatment of highly invasive OSCC, which also suggests that isoplumbagin may serve as a bioactivator/substrate for NQO1 rather than as an inhibitor.

### 2.5. Isoplumbagin Does Not Increase Oxidative Stress or DNA Fragmentation in the Highly Invasive Oral Cancer Cells

**Figure 5:**
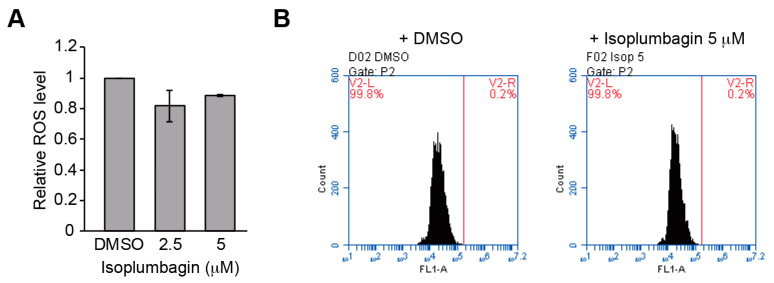
The effect of isoplumbagin on intracellular oxidative stress and DNA damage of oral squamous cell carcinoma (OSCC) cells: (**A**) OC3-IV2 cells were treated with either DMSO or isoplumbagin for 2 h. The intracellular reactive oxygen species levels were determined using the dihydroethidium (DHE) dye via flow cytometry. The mean fluorescence intensity was normalized to the DHE fluorescence in the DMSO control group. Data from two independent experiments are presented as mean ± SD. (**B**) OC3-IV2 cells were treated with either DMSO or isoplumbagin for 12 h. The DNA fragmentation was determined by deoxynucleotidyl transferase (TdT) dUTP nick end labeling (TUNEL) analysis. Representative data were from two independent experiments.

What is the molecular mechanism of isoplumbagin-induced NQO1 action on suppression of cancer cell growth and invasion? β-lapachone, which is currently under multiple phase I/II clinical trials, is known to be bioactivated by NQO1 in NQO1^high^ tumors, leading to generation of reactive oxygen species [17,18,19]. Elevated oxidative stress is correlated with tumor initiation, metastasis, and therapeutic resistance. Hence, we examined whether isoplumbagin-mediated decrease in cell viability of OC3-IV2 cells was due to the NQO1-induced intracellular oxidative stress accumulation. As shown in Figure 5A, OC3-IV2 cells showed no accumulation of reactive oxygen species with isoplumbagin treatment. Another mechanism by which unstable hydroquinone exerts cytotoxicity may be as alkylating agents like mitomycin C to induce DNA crosslinking leading to DNA damage. Therefore, the effect of isoplumbagin on DNA damage in OSCC cells was assessed by terminal deoxynucleotidyl transferase (TdT) dUTP nick end labeling (TUNEL) assays. Our results also showed no induction of DNA fragmentation by isoplumbagin in OC3-IV2 cells (Figure 5B). Considering the lower level of NQO1 in OC3-IV2 cells compared to OC3 cells, these data are not surprising. We think isoplumbagin may exert an anticancer effect in the highly invasive OSCC via a novel mechanism.

### 2.6. Isoplumbagin Regulates Mitochondrial Morphogenesis and Respiration in Highly Invasive OC3-IV2 Cells

**Figure 6:**
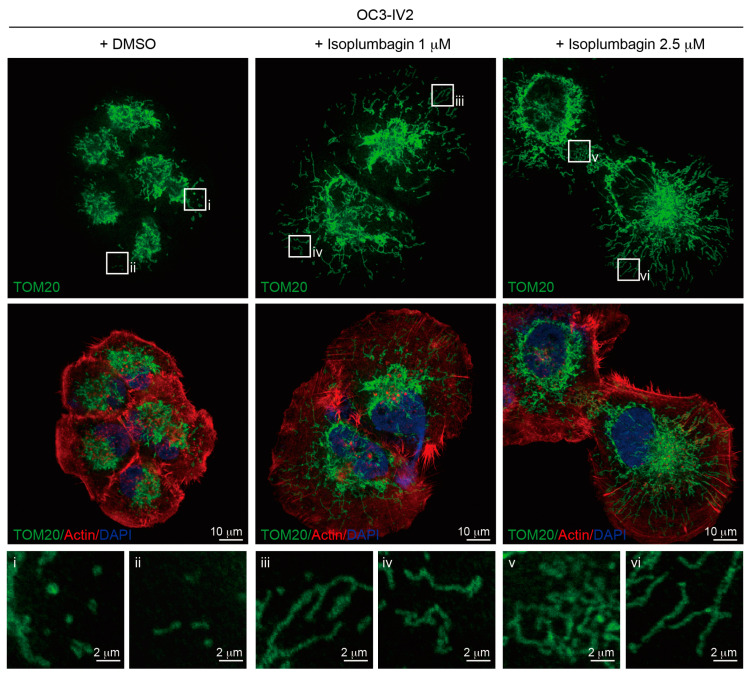
The effect of isoplumbagin on mitochondrial morphogenesis of OC3-IV2 cells: Mitochondrial morphologies of OC3-IV2 cells treated with either DMSO or isoplumbagin were determined by immunofluorescence staining using anti-TOM20 (mitochondria, green), phalloidin (actin, red), and 4′,6-diamidino-2-phenylindole (DAPI; nucleus, blue). Enlarged panels (i-vi) from the boxed area are shown in the bottom panel. Scale bar: 10 μm; 2 μm for enlarged images.

We previously reported that highly invasive oral cancer OC3-IV2 cells expressed high levels of ROS1 oncogene compared to its parental OC3 cells [9]. The upregulated ROS1 oncoprotein did not increase oxidative stress; instead, it localized to the mitochondria and increased mitochondrial fission [20]. It is thus possible that the cellular effect of isoplumbagin is associated with mitochondrial function. First, the effect of isoplumbagin on mitochondrial morphogenesis of OC3-IV2 cells was examined by immunofluorescence staining. In the isoplumbagin-treated OC3-IV2 cells, the mitochondria became elongated (presumably through increasing fusion) compared to the mostly fragmented phenotype in mock-treated cells (Figure 6). Since mitochondrial fragmentation is often associated with cancer metastasis [21] and stemness [22,23], isoplumbagin may target regulators of mitochondrial morphogenesis to exert anti-invasiveness and anti-stemness properties.

**Figure 7:**
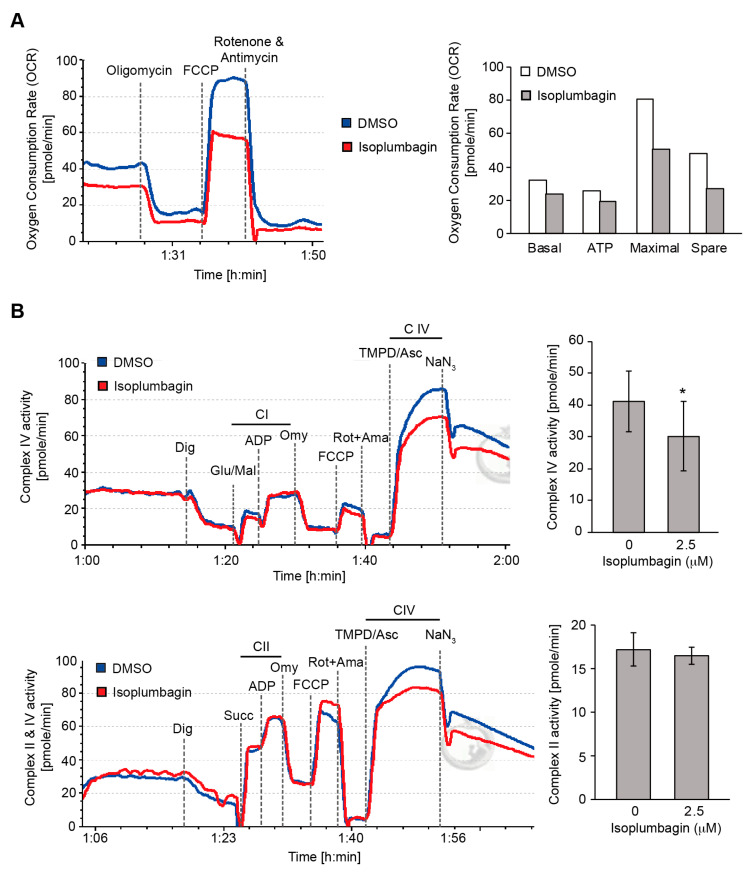
The effect of isoplumbagin on mitochondrial respiration and complex activity of OC3-IV2 cells: (**A**) the oxygen consumption rate (OCR) of OC3-IV2 cells treated with DMSO (as control) or 2.5 μM isoplumbagin were measured. This result was from one independent experiment. (**B**) OC3-IV2 cells treated with DMSO or 2.5 μM compound isoplumbagin were permeabilized with digitonin (Dig) and were added indicated substrates and inhibitors, and then mitochondria complex I/IV (upper panel) or II/IV (bottom panel) activities were determined. Glu/Mal: glutamate and malate, succinate, ADP, Omy: oligomycin, FCCP, Rot: rotenone, Ama: antimycin A, TMPD: tetramethyl-p-phenylenediamine, Asc: ascorbate and NaN_3_. Data from three independent experiments are presented as mean ± S.E.M. (* *p* < 0.05, paired Student’s *t*-test).

We examined the intact cell oxygen consumption rate (OCR) of DMSO-treated or isoplumbagin-treated OC3-IV2 cells by high-resolution respirometry using an Oxygraph-2k to determine whether isoplumbagin influenced mitochondrial respiration (OXPHOS). As shown in Figure 7A, isoplumbagin decreased basal mitochondrial OXPHOS activity. In addition, inhibiting ATP synthase activity by oligomycin was associated with a decrease of ATP generation by 24% in isoplumbagin-treated OC3-IV2 cells. Subsequently, the addition of the uncoupler carbonyl cyanide 4-(trifluoromethoxy)phenylhydrazone (FCCP), revealing the maximal capacity of the electron transport system inhibited 38% after isoplumbagin treatment, whereas spare respiratory capacity, defined as the difference between basal respiration and FCCP-induced maximal respiration, was reduced 44% by isoplumbagin. Finally, rotenone and antimycin A fully blocked respiration through inhibiting complexes I and III. These data indicate that isoplumbagin suppresses basal respiration, maximal respiration, spare respiratory capacity, and ATP production.

We directly examined how isoplumbagin reduced mitochondrial OXPHOS, the individual OXPHOS complexes’ activity following a substrate uncoupler inhibitor titration (SUIT) protocol [24] that was performed to permeabilized OC3-IV2 cells with digitonin and measured by high-resolution respirometry. The glutamate/malate-stimulated complex I linked respiration (Figure 7B, upper panel) and succinate-supported complex II linked respiration (Figure 7B, bottom panel) were not altered by isoplumbagin treatment, but tetramethyl-p-phenylenediamine (TMPD)/ascorbate-stimulated respiration of complex IV was decreased in isoplumbagin-treated OC3-IV2 cells compared to DMSO-treated cells. Taken together, these data suggest that isoplumbagin reduces mitochondrial OXPHOS through inhibiting complex IV activity. These data are consistent with our findings that the highly invasive OSCC correlated with enhanced mitochondrial fission, biogenesis, and cell metabolic plasticity [20]. 

## 3. Materials and Methods 

### 3.1. Antibodies and Reagents

Anti-TOM20 (#sc-11415, 1:200 for immunofluorescence staining) and anti-NQO1 (#sc-32793, 1:1000 for immunoblotting) were purchased from Santa Cruz Biotechnology (Santa Cruz, CA, USA). Anti-glyceraldehyde 3-phosphate dehydrogenase (GAPDH) (#10494-1-AP, 1:20,000 for immunoblotting) was purchased from Proteintech (Rosemont, IL, USA). Alexa Fluor 488 (#A11001) or Alexa Fluor 700 (#A21036)–conjugated secondary antibodies, Alexa Fluor 488–conjugated phalloidin (#A12379), and 4′,6-diamidino-2-phenylindole (DAPI) (#1306) were obtained from Invitrogen (Carlsbad, CA, USA). IRDye800CW-labeled anti-rabbit secondary antibody (#926-32211) was purchased from LI-COR Biosciences (Lincoln, NE, USA). Isoplumbagin (5-hydroxy-3-methyl-1,4-naphthoquinone) was acquired from AKos GmbH (Steinen, Germany). Oligomycin (#75351), carbonyl cyanide 4-(trifluoromethoxy)phenylhydrazone (FCCP) (#C2920), rotenone (#R8875), antimycin A (#A8674), lactobionic acid (#153516), taurine (#T8691), digitonin (#D141), glutamate (#G8415), malate (#M1000), succinate (#S3674), adenosine 5′-diphosphate sodium salt (ADP) (#A2754), TMPD (#T3134), and ascorbate (#A4034) were purchased from Sigma-Aldrich (St Louis, MO, USA).

### 3.2. Cell Culture

Human oral cancer cell line OC3-IV2 cells have been described [9,25,26] and were cultured in a 1:1 ratio of Dulbecco’s modified Eagle medium (DMEM; Invitrogen) and keratinocyte serum-free medium (KSFM; Invitrogen) containing 10% (*v*/*v*) fetal bovine serum (Invitrogen), 1% (*v*/*v*) l-glutamine (Invitrogen), and 1% (*v*/*v*) antibiotic-antimycotic (Invitrogen). Human primary glioblastoma cell line U87 cells were obtained from the American Type Culture Collection (ATCC, Manassas, VA, USA) and grown in DMEM medium supplemented with 10% (*v*/*v*) fetal bovine serum, 1% (*v*/*v*) Minimum Essential Medium (MEM) non-essential amino acids solution (Invitrogen), and 1% (*v*/*v*) penicillin/streptomycin (Invitrogen). Human prostate cancer cell line PC3 cells, human cervical cancer cell line HeLa cells, and 293T cells were obtained from the ATCC and maintained in DMEM medium supplemented with 10% (*v*/*v*) fetal bovine serum, 1% (*v*/*v*) l-glutamine, and 1% (*v*/*v*) antibiotic-antimycotic. Human non-small cell lung carcinoma cell line H1299 cells were obtained from the ATCC and were cultured in Roswell Park Memorial Institute (RPMI) 1640 medium (Invitrogen) containing 10% (*v*/*v*) fetal bovine serum, 1% (*v*/*v*) l-glutamine, and 1% (*v*/*v*) antibiotic-antimycotic. Cells were maintained in a humidified atmosphere containing 5% CO_2_ at 37 °C, and the culture medium was replaced every 2 days.

### 3.3. MTT Assays and Boyden Chamber Assays

Cytotoxicity was determined using the 3-(4,5-dimethylthiazol-2-yl)-2,5-diphenyltetrazolium bromide (MTT; Sigma-Aldrich) assays; 3 × 10^3^ cells/well were seeded in 96-well plates [27]. After incubation for 24 h, cells were treated with isoplumbagin at various concentrations and incubated for a further 48 h. At the end of treatment, MTT was added to each well. After incubation for 3 h, medium was removed and DMSO was added to each well to dissolve reduced MTT product formazan. The absorbance of dissolved formazan was measured at 550 nm wavelengths by a spectrophotometer. The ability to invade cell was evaluated by Boyden chamber assay. Cells were harvested by trypsin and resuspended in a serum-free medium with 0.1% (*w*/*v*) bovine serum albumin. Then, 200 mL of cell suspension (2 × 10^5^ cells) was seeded into the upper chamber of SPLInsert with Polyethylene terephthalate (PET) membrane (pore size: 8.0 μm) (SPL Lifesciences, Korea) pre-coated with matrigel (BD Biosciences, San Jose, CA). The bottom well of a 24-well plate was filled with cell medium containing 10% (*v*/*v*) fetal bovine serum with or without isoplumbagin. DMSO or isoplumbagin were separately added in the suspension of cells in the upper chamber. After incubation for 24 h, the cells that migrated to the bottom side of the PET membrane were fixed with 4% paraformaldehyde and stained with crystal violet. Photos of stained cells on the underside of PET membrane were taken using Zeiss Observer Z1 microscope (Zeiss, Jena, Germany). The number of cells was counted using Image J. Relative invasion ability was calculated as cell number per area.

### 3.4. In Vivo Xenograft Mice Model

All procedures were performed according to approved National Tsing Hua University Institutional Animal Care and Use Committee (approval number: 10602, approval date: 2017/02/17) and National Chiao Tung University Institutional Animal Care and Use Committee (approval number NCTU-IACUC-108032, approval date: 2019/07/22) protocols. Briefly, in an oral orthotopic xenograft CB17/lcr-*Prkdc^scid^*/CrlNarl mice model, 1 × 10^6^ OC3-IV2 cells were harvested and resuspended in 100 μL phosphate-buffered saline (PBS). The cells were injected through the oral buccal mucosa into six-week-old male CB17/lcr-*Prkdc^scid^*/CrlNarl mice (National Laboratory Animal Center, Taiwan). When the tumor size in oral buccal reached approximately 25 mm^3^ at 14 days after inoculation of the OC3-IV2 cells, mice were randomly assigned into two groups: the vehicle (DMSO) control group (*n* = 13) and the 2 mg/kg isoplumbagin group (*n* = 10). Treatments were given via intraperitoneal injection once every 3 days in the morning for 2 weeks. During this period, mice weights and tumor volumes (length × width^2^/2) were recorded every 3 days. At the end of the study (on day 18), the mice were sacrificed by carbon dioxide asphyxiation.

### 3.5. Molecular Docking

To perform molecular docking of NQO1 and isoplumbagin, the crystallographic structure of NQO1 (PDB code 2F1O) and isoplumbagin (PubChem CID: 375105) was applied. The complex model of NQO1 and isoplumbagin was performed using PyRx (v.0.8) [28]. The structural model representations and docked orientations were generated by PyMOL (v.2.3.4; DeLano Scientific, San Carlos, CA, USA) and Ligplot^+^ (v.2.2) [29].

### 3.6. NQO1 Activity Assays

The NQO1 activity assays kit (#ab184867) was purchased from Abcam (Cambridge, MA, USA). According to the manufacturer’s instructions, OC3-IV2 cells were lysed with 1X extraction buffer containing 1 mM phenylmethylsulfonyl fluoride for 15 min on ice, and then centrifuged at 17,000 × *g* at 4 °C for 20 min. The supernatant was collected, and then, the protein concentration of each sample was determined by bicinchoninic acid assays (Santa Cruz Biotechnology). A diluted sample and reaction buffer were added into 96-wells plate containing menadione or isoplumbagin with cofactor NADH and water-soluble tetrazolium salt (2-(4-iodophenyl)-3-(4-nitrophenyl)-5-(2,4-disulfophenyl)-2H-tetrazolium, a highly sensitive tetrazolium reagent). The absorbance of reduced water-soluble tetrazolium salt was measured at 450 nm wavelengths by a spectrophotometer.

### 3.7. Immunobloting

Cell lysates of OC3, OC3-IV2, H1299, and 293T cells were lysed by radioimmunoprecipitation assay buffer [50 mM Tris, pH 7.5, 1% (*v*/*v*) Triton X-100, 150 mM NaCl, and 2 mM ethylene glycol tetraacetic acid (EGTA)]containing 1 mM sodium orthovanadate, 1 mM phenylmethylsulfonyl fluoride, 10 ng/mL leupeptin, and 10 ng/mL aprotinin. The protein concentration of each sample was determined by the bicinchoninic acid assays, and samples were separated by SDS-PAGE, followed by immunoblotted with the indicated primary antibodies and the IRDye-conjugated secondary antibody. The immunoblots were detected using the Odyssey infrared imaging system (LI-COR Biosciences).

### 3.8. Reactive Oxygen Species Assays

Cells were treated with DMSO or isoplumbagin for 2 h and then harvested with trypsin. Equal numbers of cells (5 × 10^5^ cells/mL) were incubated with 5 μM of dihydroethidium (DHE; Invitrogen), a cytoplasmic superoxide indicator, for 30 min at room temperature away from light. The intracellular reactive oxygen species levels were measured by BD Accuri™ C6 flow cytometer (BD Biosciences, San Jose, CA). Histograms of 20,000 events were analyzed, and DHE fluorescence was measured by filter FL-2 (585 nm). The mean fluorescence intensity was calculated by BD Accuri C6 Software.

### 3.9. TUNEL Assays

Cells were treated with DMSO or isoplumbagin for 12 h. After fixing with 1% (*v*/*v*) paraformaldehyde for 15 min, cells were washed with PBS two times and added to 70% (*v*/*v*) ethanol for 30 min on ice. After that, cells were stained by the usage of TUNEL staining kit (Abcam, #ab66108, Cambridge, MA, USA) according to the manufacturer’s instructions. Briefly, cells were incubated with DNA labeling solution for 60 min at 37 °C, followed by the addition of PI/RNase A solution for 30 min. TUNEL-labeled cells were evaluated by BD Accuri™ C6 flow cytometer, and the data were analyzed using BD Accuri C6 Software.

### 3.10. Immunofluorescence Staining

Cells were fixed by 4% (*v*/*v*) paraformaldehyde and permeabilized by 0.1% (*v*/*v*) Triton X-100. Cells were incubated in blocking buffer containing 1% bovine serum albumin. After blocking, cells were incubated overnight with indicated primary antibodies against TOM20 (a mitochondria marker) and then incubated with Alexa Fluor-conjugated secondary antibodies. The nucleus was stained with DAPI, actin was stained with phalloidin, and cells were mounted using Prolong Gold reagent. Images were taken by LSM800 confocal microscopes (Zeiss).

### 3.11. High.-Resolution Respirometry

Oxygen consumption rate in intact cells suspension was measured by the Oxygraph-2k system (Oroboros Instruments, Innsbruck, Austria). DMSO or isoplumbagin-treated cells were detached from the plate by trypsinization and suspended at 5 × 10^5^ cells/mL in DMEM/KSFM medium supplemented with 10% fetal bovine serum into the O2k chambers. The oxygen consumption rate of DMSO or isoplumbagin treated cells samples were measured by adding 0.5 μM oligomycin, 0.5 μM FCCP, 1 μM rotenone, and 1 μM antimycin A. For cell permeabilization and measurement of respiration in permeabilized cells, 5 × 10^5^ cells suspended in 2 mL MiR05 buffer (110 mM D-sucrose, 0.5 mM EGTA, 3.0 mM MgCl_2_, 60 mM lactobionic acid, 10 mM KH_2_PO_4_, 20 mM taurine, 20 mM HEPES, 1 g/L bovine serum albumin, and pH 7.1) were added in O2k chambers. After cells had stabilized at routine respiration, plasma membrane permeabilization was performed by adding 5 μM digitonin, and then, O2 consumption rate was measured in response to sequential additions of 10 mM glutamate and 2 mM malate or 10 mM succinate, followed by 5 mM ADP, 0.5 mM oligomycin, 0.5 μM FCCP, 1 μM rotenone, 1 μM antimycin A, 0.1 mM TMPD, 0.4 mM ascorbate, and 5 mM NaN_3_.

### 3.12. Statistical Analysis

Statistical analysis of results was carried out using the Student’s *t*-test, paired Student’s *t*-test, or one-way ANOVA (Tukey’s Test). Values reflect the mean ± SD of data obtained from two independent experiments or the mean ± S.E.M. of data obtained from at least three independent experiments. Statistical significance was defined as *p* < 0.05.

## 4. Conclusions

These findings reveal that isoplumbagin is an anticancer chemical against human oral squamous cell carcinoma, glioblastoma, non-small cell lung carcinoma, and prostate and cervical cancers. The therapeutic effect of isoplumbagin is supported by the OSCC xenograft in vivo results. Our mechanistic study identifies a novel mechanism of the isoplumbagin drug effect through acting as a NQO1 substrate and through modulating mitochondrial morphogenesis and respiration.

## Supplementary Materials

Supplementary materials can be found at https://www.mdpi.com/1422-0067/21/12/4378/s1. Supplementary Figure S1. The chemically synthesized isoplumbagin and HPLC analysis. Supplementary Figure S2. Scheme of two electron reductions of isoplumbagin by NQO1 generating the hydroquinone. Supplementary Figure S3. The relative NQO1 expression and the effect of dicumarol on survival of OSCC cells: Proliferation of OC3-IV2 cells treated with dicoumarol for 48 h was measured with the MTT assay. Data from three independent experiments are presented as mean ± S.E.M.

## Abbreviations

OSCC	Oral squamous cell carcinoma
NQO1	NAD(P)H dehydrogenase quinone 1
OCR	Oxygen consumption rate
OXPHOS	Mitochondrial respiration
