# Supplementary material for: Discovery of Isoplumbagin as a Novel NQO1 Substrate and Anti-Cancer Quinone
Source: Int J Mol Sci. 2020 Jun 19;21(12):4378. doi: 10.3390/ijms21124378 (PMC7352187; doi:10.3390/ijms21124378)
Supplement: Supplementary file 1 [file ijms-21-04378-s001.zip › Supplementary Figure 1.pdf]

Creation date: 2019.06.18 Expiration date: 2023.09.16 Lot number: F307035G

2019.6.27 YQ

4°C

Certificate Of Analysis

FCH Group

14017, Chernigiv, Ukraine
Schorsa Str. 66/26

Tel/Fax 38 (044) 495 88 16
E-mail: info@fchgroup.net

Isoplumbagin

Product Name : 8-hydroxy-2-methyl-1,4-dihydronaphthalene-1,4-dione

Cat Number(s): FCH843524

Product labeling: AKOS006277326

Molecular Formula : C11H8O3

CAS Number: 14777-17-4

Molecular Weight: 188.1794

Storage Temperature, °C: +4°C

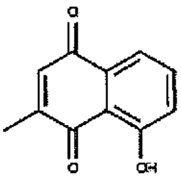

Tests	Specifications	Results
State:	powder	powder
Colour:	brown	brown
Melting point, °C:	N/A	139
Purity (1H NMR)	95%	95%, conforms with structure, R1478117.WMF
GCMS	95%	97.84%, conforms with structure, R1478117.PDF

The product is intended for laboratory use only. The product should be handled and used by trained professional personnel. The responsibility for the safe handling and use of this product rests solely with the buyer and/or user. The data and information as stated was furnished by the manufacturer of the product. The information provided in this certificate pertains only to the lot number specified.

R1478117

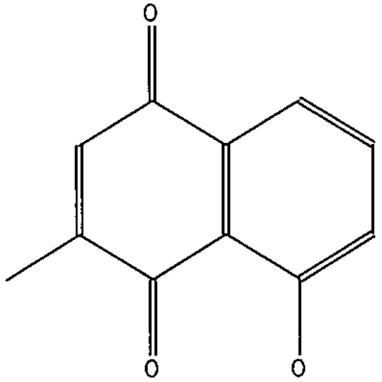

R1478117 C11H8O3 188.18

FCH843524

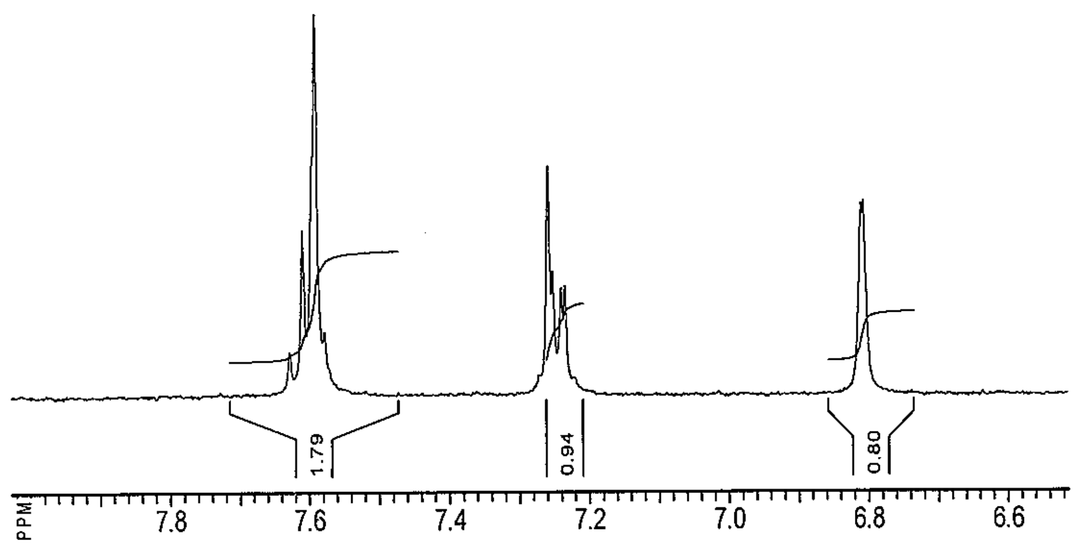

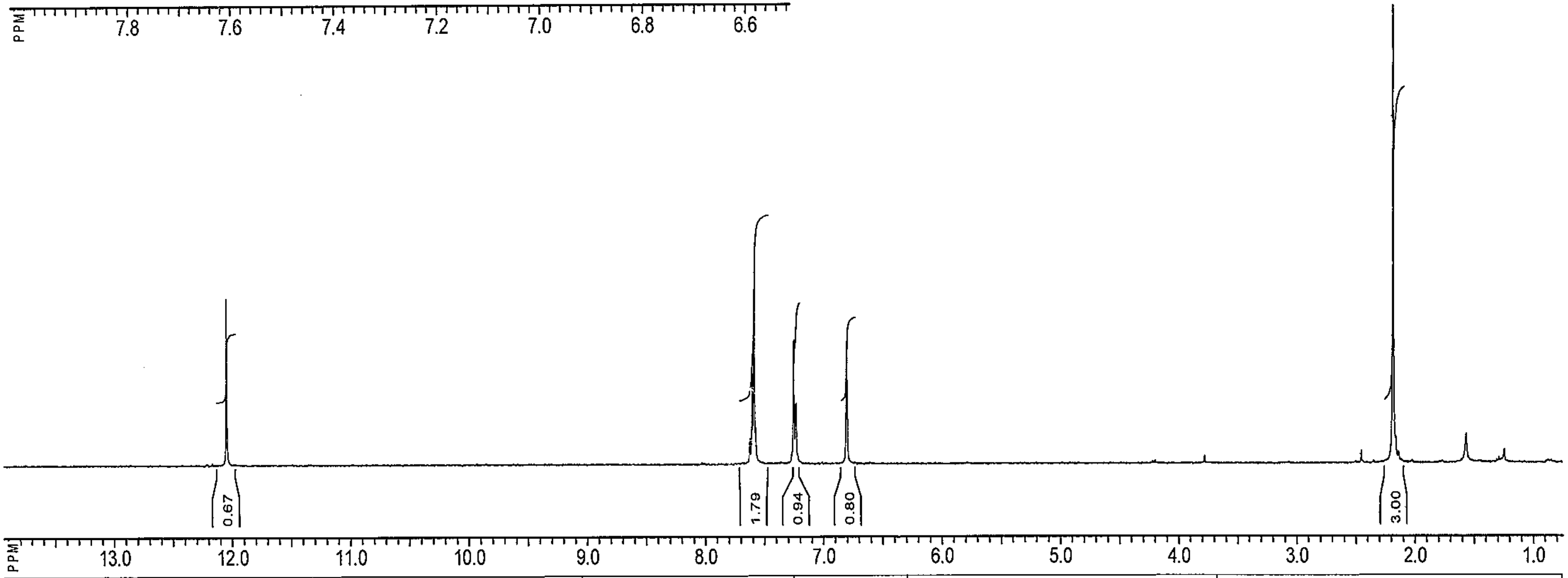

File name: R1478117	Operator: Zhelezniak Angelika	SF: 399.9781 MHz	NSC: 0	PW: 7.10 usec, RG: 32	SI: 32768

Library Search Report

Data File : C:\MSDCHEM\1\DATA\06_18\R1478117.D Vial: 4
Acq On : 18 Jun 2019 12:01 Operator:
Sample : R1478117 Inst : Instrumen
Misc : CH3CN Multiplr: 1.00
Sample Amount: 0.00

MS Integration Params: autoint1.e
Method : C:\MSDCHEM\1\METHODS\UNIVERS.M (Chemstation Integrator)
Title :

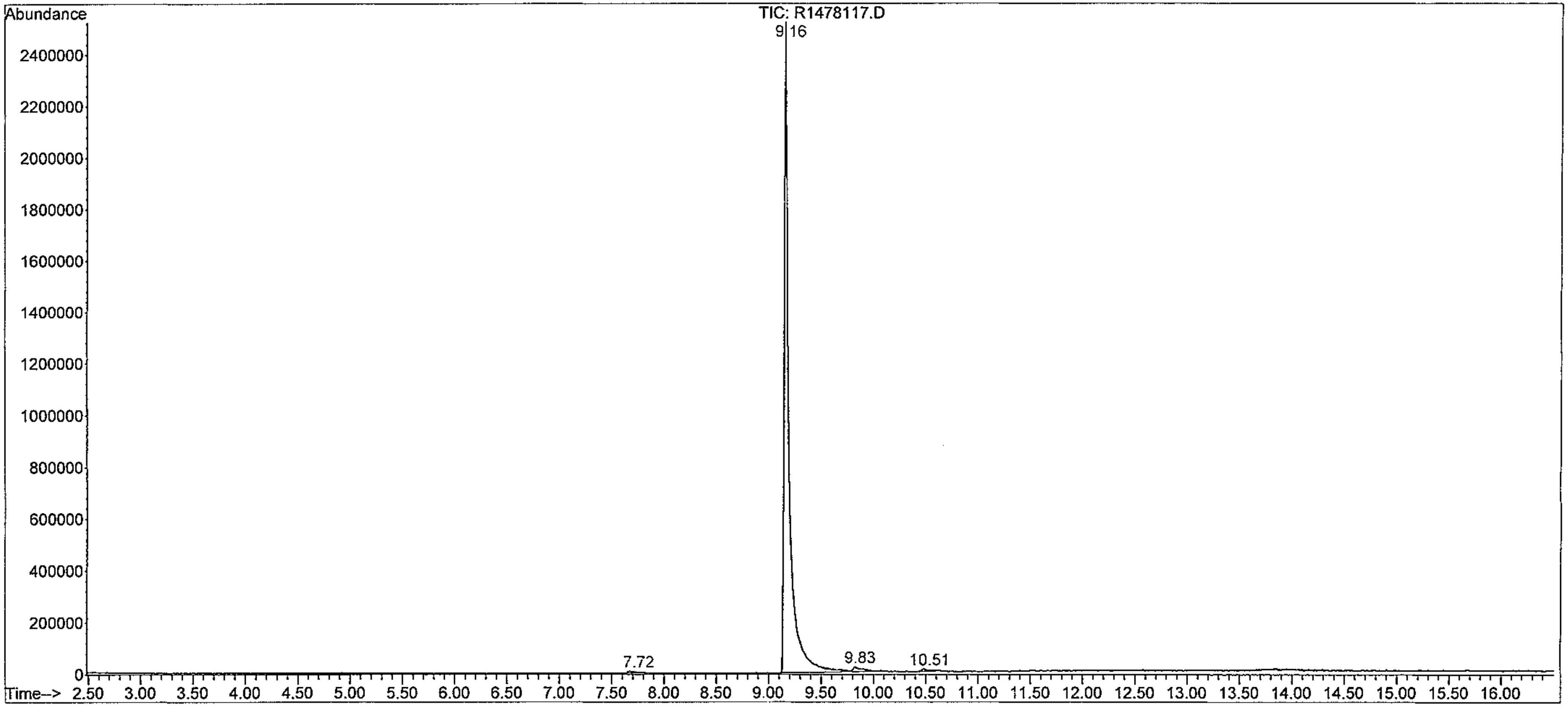

Unknown Spectrum based on Apex

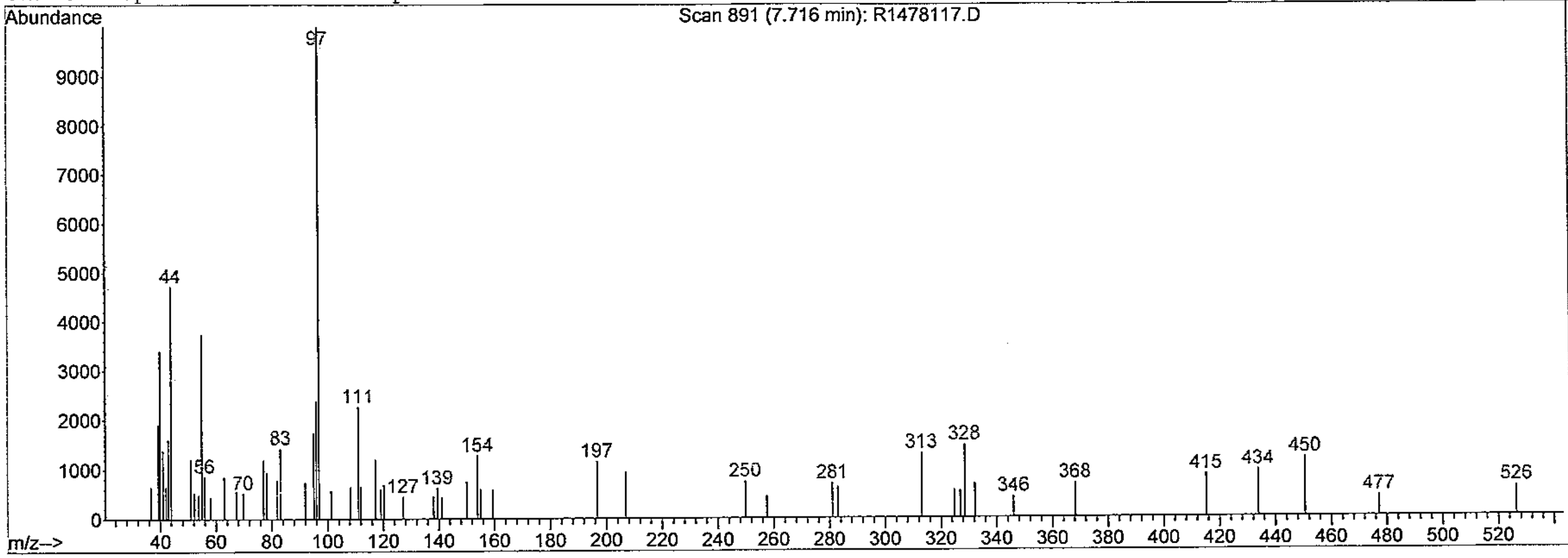

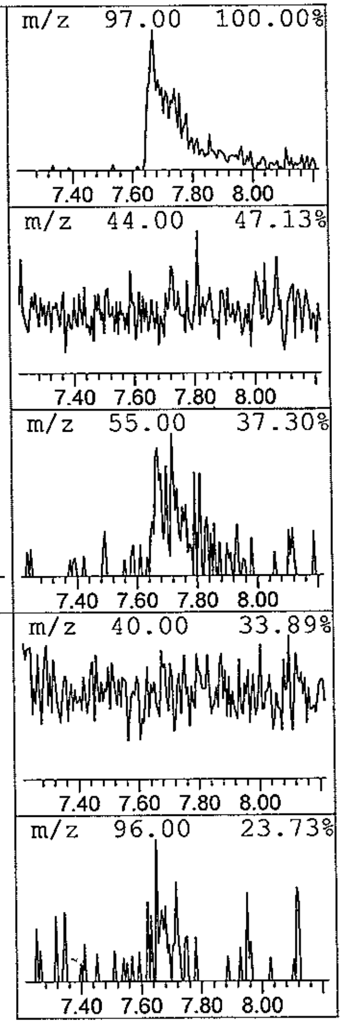

Data File: C:\MSDCHEM\1\DATA\06_18\R1478117.D
Sample : R1478117

Peak Number: 1 at 7.72 min Area: 260771 Area % 0.32

CTDatabase\EMPTY [illegible] Ref# CAS# Qual

Unknown Spectrum based on Apex

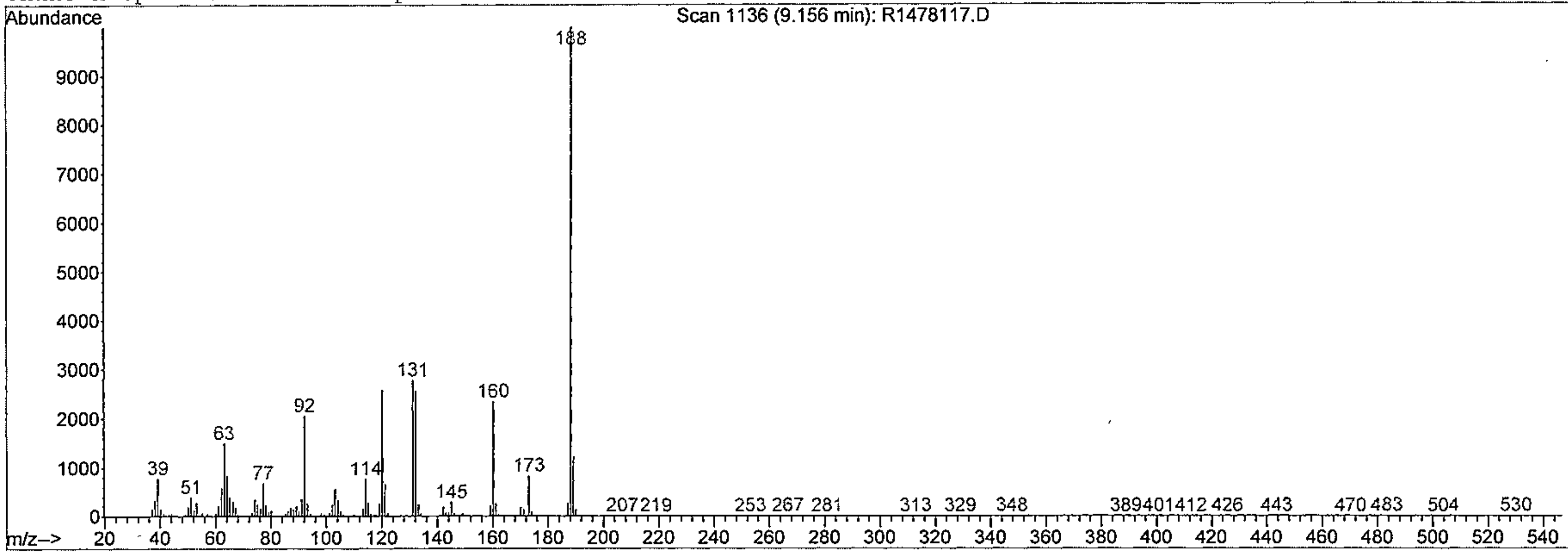

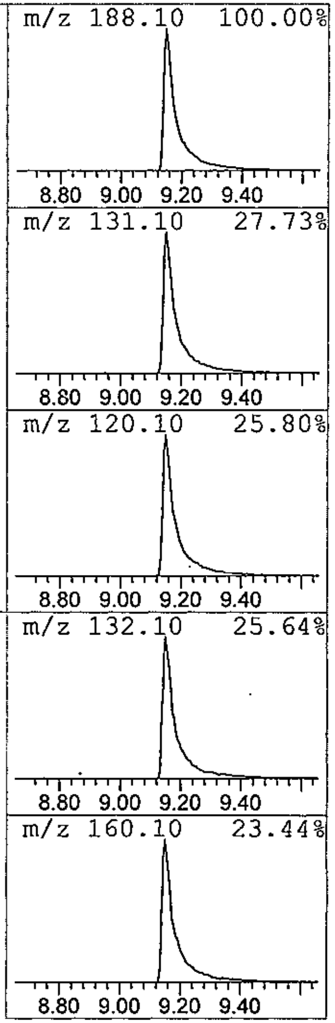

Data File: C:\MSDCHEM\1\DATA\06_18\R1478117.D
Sample : R1478117

Peak Number: 2 at 9.16 min Area: 79088773 Area % 97.84

CThèDaSabase\EMPTYfrom eaNb libsawere retrieveRef# CAS# Qual

Unknown Spectrum based on Apex

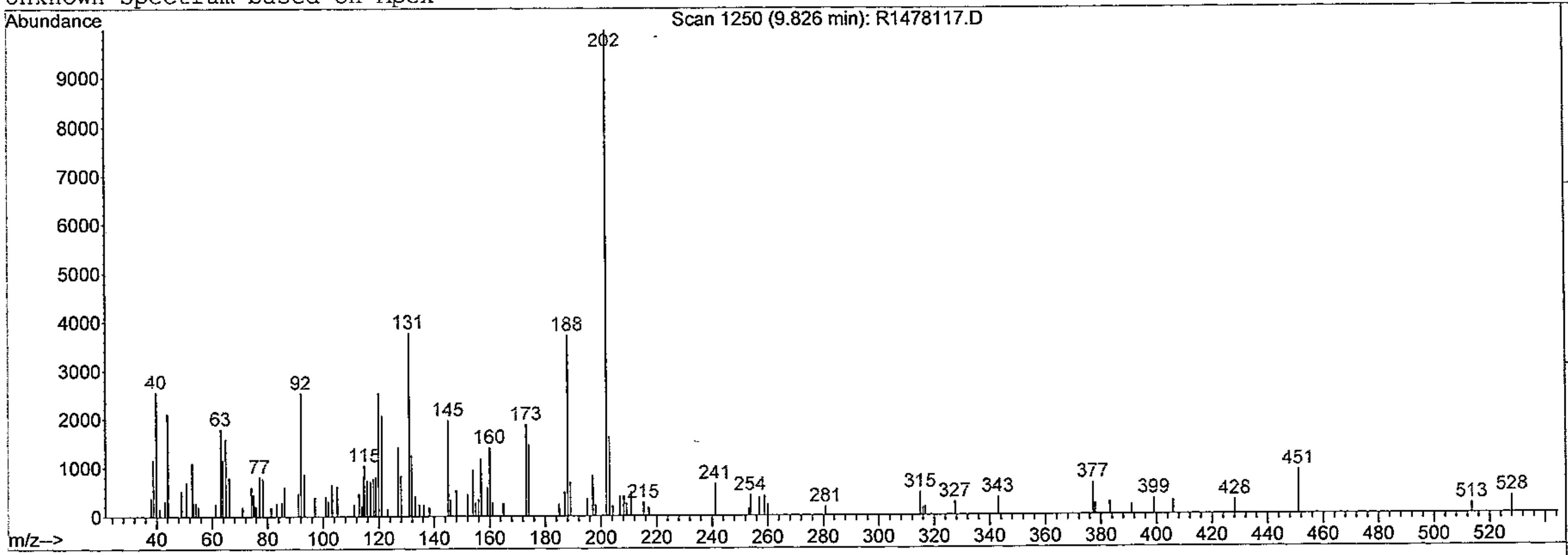

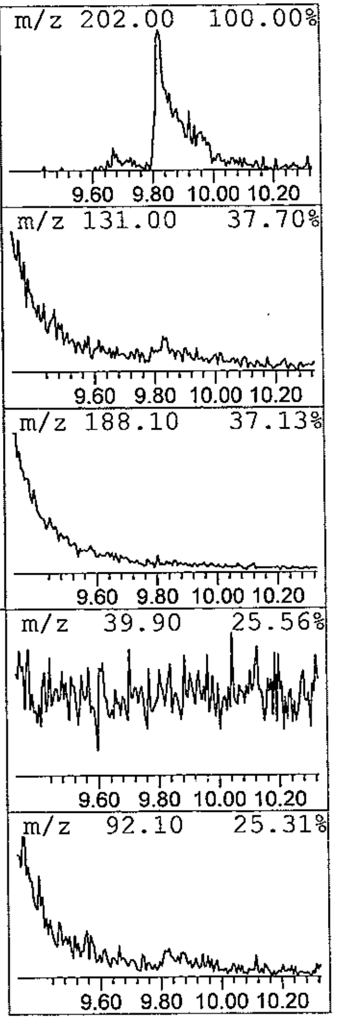

Data File: C:\MSDCHEM\1\DATA\06_18\R1478117.D
Sample : R1478117

Peak Number: 3 at 9.83 min Area: 1104908 Area % 1.37

CThDaBabase\EMPSYfIom eaNb libsawgre retrieveRef# CAS# Qual

Library Search Report - Chemstation Integrator

Unknown Spectrum based on Apex

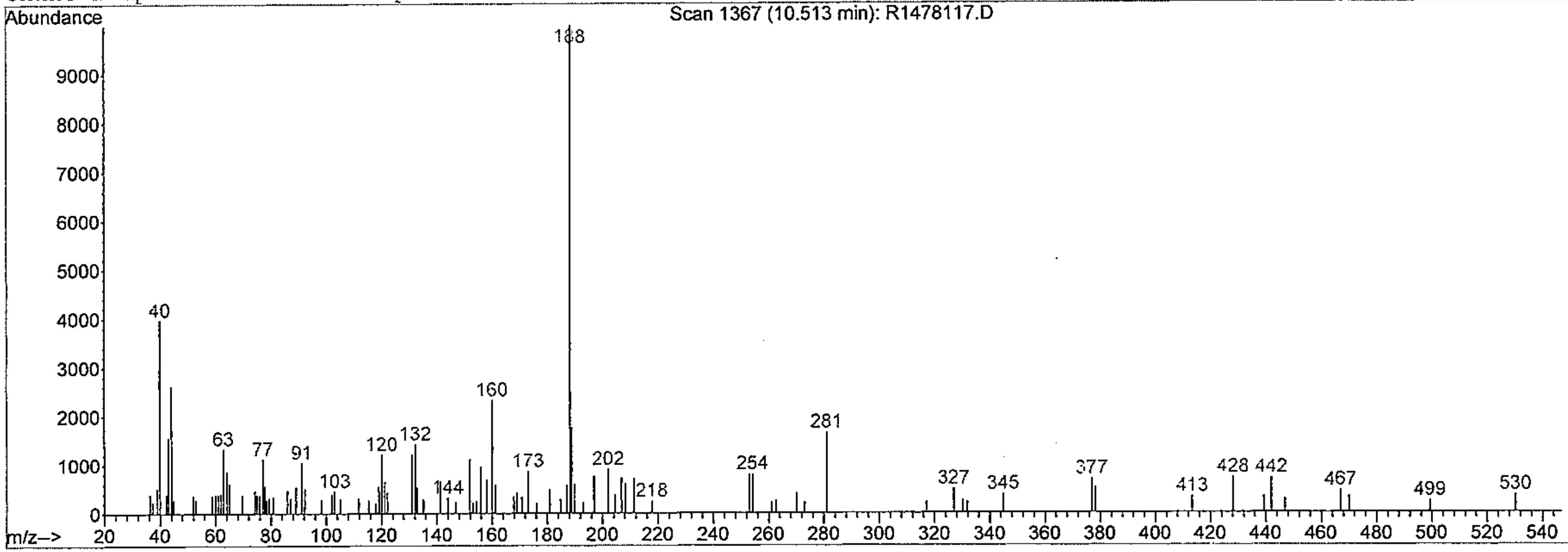

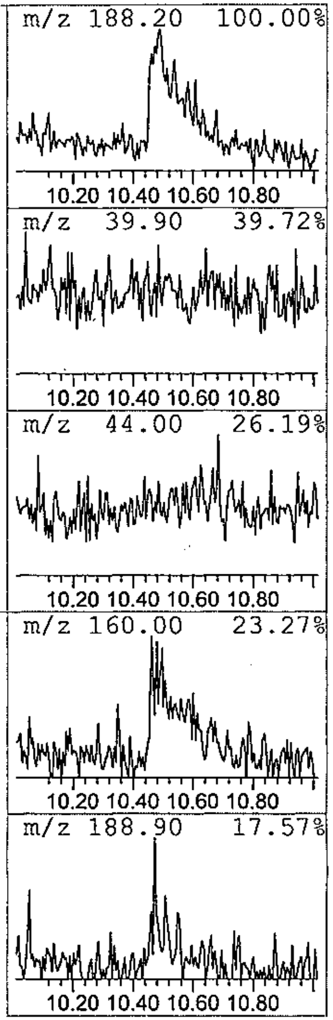

Data File: C:\MSDCHEM\1\DATA\06_18\R1478117.D
Sample : R1478117

Peak Number: 4 at 10.51 min Area: 377967 Area % 0.47

CThDaBabese\EMPSYfLom eaNb hibsawgre retrieveRef# CAS# Qual

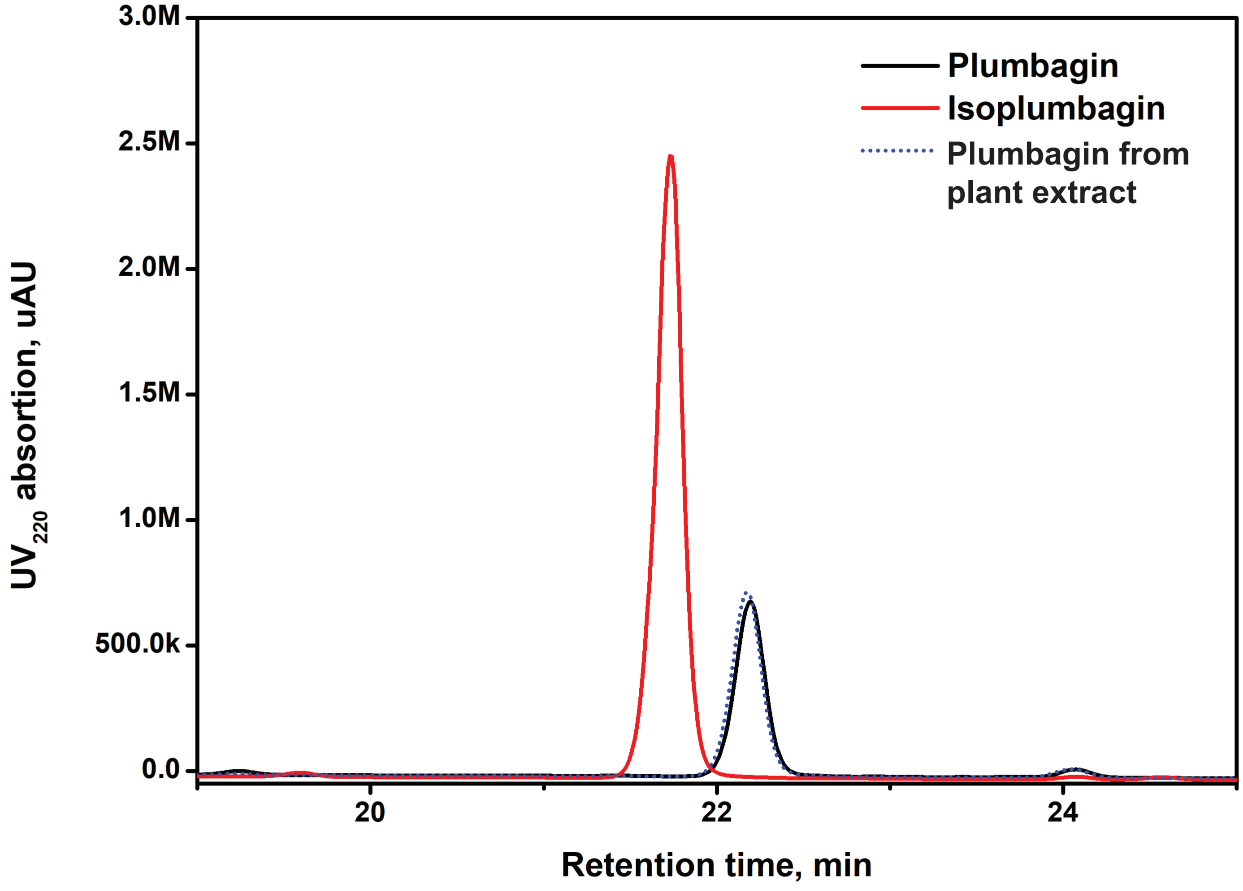
3.0M
2.5M
2.0M
1.5M
1.0M
500.0k
0.0
20
22
24
UV220 absorption, uAU
Retention time, min
Plumbagin
Isoplumbagin
Plumbagin from plant extract
