# Supplementary material for: Discovery of Isoplumbagin as a Novel NQO1 Substrate and Anti-Cancer Quinone
Source: Int J Mol Sci. 2020 Jun 19;21(12):4378. doi: 10.3390/ijms21124378 (PMC7352187; doi:10.3390/ijms21124378)

Supplementary Figure 2. Scheme of two electron reduction of isoplumbagin by NQO1 generates the hydroquinone.


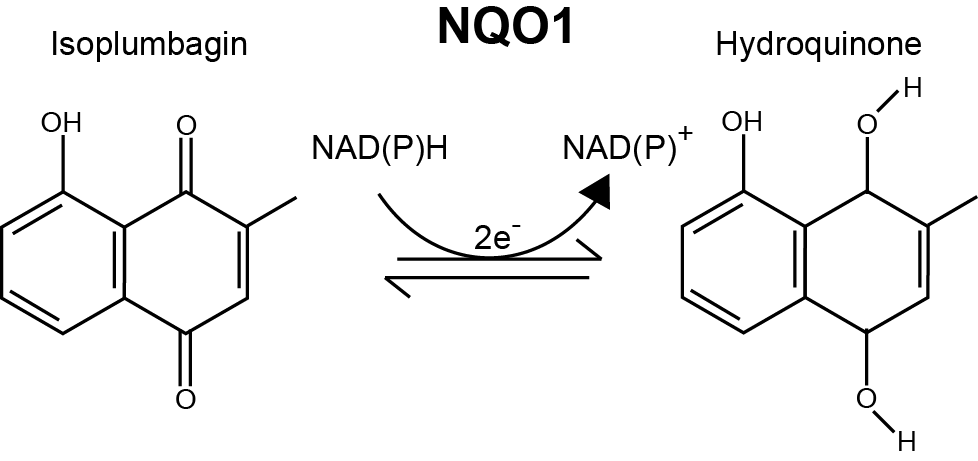


**Supplementary Figure 3.** The relative NQO1 expression and the effect of dicumarol on survival of OSCC cells. Proliferation of OC3-IV2 cells treated with dicoumarol for 48 h was measured with the MTT assay. Data from three independent experiments are presented as mean ± S.E.M.


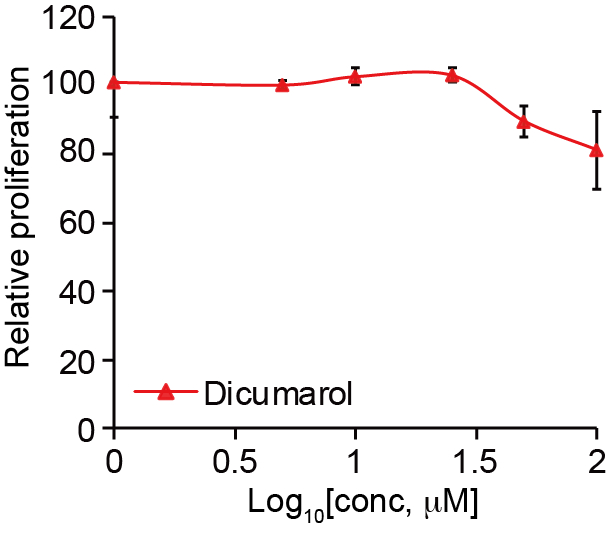

Supplement: Supplementary file 1 [file ijms-21-04378-s001.zip › Supplementary Figure 2-3.docx]
